# Positive Association Between the Cardiometabolic Index and the Risk of Male Biochemical Androgen Deficiency in Adults

**DOI:** 10.1002/kjm2.70024

**Published:** 2025-04-09

**Authors:** Shuai‐Shuai Mao, Wei Huang, Jia‐Qing Luo

**Affiliations:** ^1^ Endocrine Department Changxing People's Hospital Huzhou Zhejiang China; ^2^ General Surgery Changxing People's Hospital Huzhou Zhejiang China

**Keywords:** association, biochemical androgen deficiency, cardiometabolic index, cross‐sectional study, NHANES

## Abstract

Metabolic disorders are associated with testosterone deficiency, and the cardiometabolic index (CMI) is a recently identified metabolic indicator. The relationship between male biochemical androgen deficiency (MBAD), a precursor to testosterone deficiency, and CMI remains unclear. In this cross‐sectional study, we analyzed data from the National Health and Nutrition Examination Survey (NHANES) 2013–2016 to investigate the relationship between MBAD and CMI in men. This study included 1229 participants; among which, 209 participants had MBAD. Machine learning models identified that the importance of CMI on MBAD was in the top three. After adjusting for all covariates, we found a positive association between CMI and MBAD. Restricted cubic spline (RCS) curves validated this association both in age and body mass index subgroups. Trend regression showed that participants with a higher CMI tended to have a higher risk of MBAD. The positive association between CMI and MBAD persisted after multiple interpolations, validating the robustness of the results. Altogether, this study suggests that CMI exhibits a stable positive relationship with MBAD.

## 1 Introduction

Testosterone, the primary male sex hormone in humans [1], plays a crucial role in the male reproductive system alongside C19 androgens. It contributes to the development and maintenance of male secondary sex characteristics and influences skeletal muscle, erythropoiesis, and iron metabolism [2, 3]. Some studies have shown that testosterone deficiency (TD) is strongly associated with the development of metabolic disorders, such as diabetes, cardiovascular disease, chronic kidney disease, nonalcoholic fatty liver disease (NAFLD), osteoporosis, and Alzheimer's disease [4, 5, 6, 7]. By 2025, an estimated 6.5 million men in the United States are expected to develop symptomatic TD [8]. Male biochemical androgen deficiency (MBAD) is defined as a testosterone level of < 300 ng/mL without corresponding physiological or clinical manifestations, which is considered the pre‐TD stage [9].

The cardiometabolic index (CMI) is a recently identified metabolic index for assessing cardiovascular health and metabolic risk [10]. It combines the waist‐to‐height ratio (WHtR) with the levels of triglycerides (TGs) and high‐density lipoprotein cholesterol (HDL‐C) [11]. In addition to cardiovascular disease and diabetes, other diseases have been associated with CMI. In a study involving 374 Chinese patients with a normal body mass index (BMI), the CMI of the hyperuricemia group was found to be significantly higher than that of the control group, and a positive correlation was observed between CMI and serum uric acid levels (*r* = 0.425). Specifically, the prevalence of hyperuricemia increased with an increase in CMI [12]. In another study, CMI was positively related to the risk of NAFLD in various models. Similar results were observed for liver fibrosis, and CMI demonstrated good predictive ability for both diseases [13]. Furthermore, a large‐scale retrospective cross‐sectional study from Japan showed that the CMI of men was higher than that of women. The CMI of men was higher in the middle age group, whereas that of women increased with age [14].

CMI is associated with several human diseases and has emerged as an important predictor of these diseases. However, the relationship between CMI and MBAD remains unclear. In this cross‐sectional study, we investigated this relationship using data from the National Health and Nutrition Examination Survey (NHANES) and examined the potential role of CMI in the development of MBAD.

## 2 Methods

### 2.1 Data Sources and Study Population

Data were obtained from NHANES 2013–2014 and 2015–2016. Sex steroid hormone testing was performed in these two survey cycles; therefore, total testosterone (TT) data were available.

**FIGURE 1:**
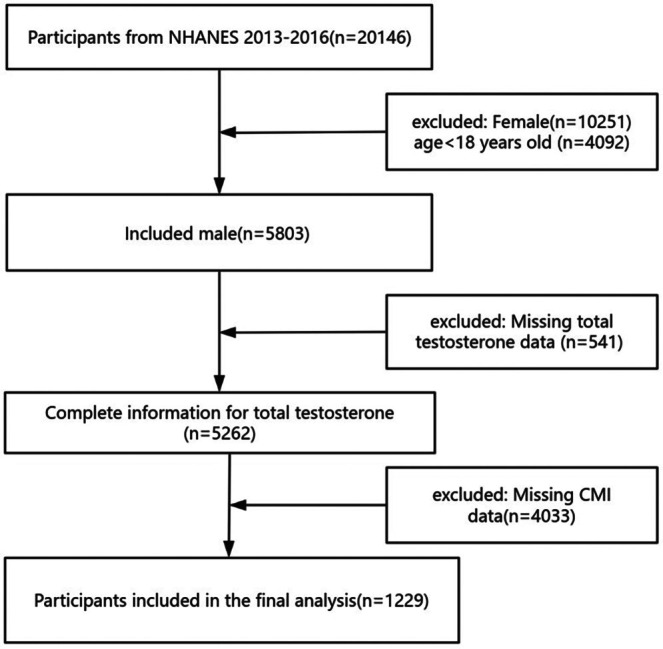
Flowchart of study population inclusion.

Male participants aged ≥ 18 years were enrolled in this study. Participants with missing TT and CMI data were excluded. Eventually, a total of 1229 participants were included (Figure 1). The protocol of NHANES was approved by The National Center for Health Statistics (NCHS) Research Ethics Review Board, and all participants provided written informed consent.

### 2.2 Representative Nature of the Study

In this study, only 21.18% of adult men from NHANES 2013–2016 had available CMI data. To verify the representativeness of the study population, we calculated the sample size using the following formula:

(1) $$N=\frac{Z_{1-\alpha /2}^{2}p\left(1-p\right)}{d^{2}}$$

In the above mentioned formula, *α* value is 0.05, *Z* value is 1.96, *d* represents the tolerance error (0.05), and *p* represents the prevalence rate of MBAD. The prevalence of MBAD has been reported to be 26.02% in previous studies [3]. Based on the above mentioned Formula (1), the required sample size for this study was 296. The total number of participants included in this study was 1229, which is greater than the required sample size. Therefore, the study population was considered a representative sample.

CMI was calculated from a combination of several indicators; therefore, the absence of any indicator would result in the failure of CMI calculation. As the indicators used to calculate CMI were missing at random, the study participants were considered a representative sample as a whole.

### 2.3 Calculation of CMI

As described in a previous study, CMI was calculated using three indicators, namely, WHtR, TG levels, and HDL‐C levels. WHtR is the ratio of waist circumference (WC, cm) to body height (BH, cm). The formula used to calculate CMI is as follows:

(2) $$\text{WHtR}=\frac{\mathrm{WC}\;\left(\mathrm{cm}\right)}{\mathrm{BH}\;\left(\mathrm{cm}\right)}$$

(3) $$\mathrm{CMI}=\frac{\mathrm{TG}\;\left(\text{mmol}/L\right)}{\mathrm{HDL}-C\;\left(\text{mmol}/L\right)}\times \text{WHtR}$$

### 2.4 Primary Outcome and Its Measurement

The primary outcome was MBAD, which is defined as a serum TT level of < 300 ng/dL according to the American Urological Association guidelines [9]. In NHANES, TT levels were measured using precise isotope dilution liquid chromatography and tandem mass spectrometry at a single time point in the morning, afternoon, or evening.

### 2.5 Covariates

Potential variables influencing the association between CMI and MBAD were categorized into three dimensions demographic characteristics, lifestyle behaviors, and health status.

Demographic variables included age, race, marital status, education level, BMI, and poverty‐income ratio. Marital status was classified as M1 (never married, separated, or living with a partner), M2 (married), and M3 (widowed or divorced). BMI was calculated by dividing weight by height squared (kg/m^2^).

Lifestyle behaviors included drinking status, sleep problems (sleep duration, trouble sleeping), smoking, and sexual behaviors. For drinking status, participants were classified as non‐drinkers (< 12 drinks/lifetime), former drinkers (> 12 drinks/year but no drinks in the previous year or > 12 drinks/lifetime but no drinks in the previous year), light drinkers (< 2 drinks/day), moderate drinkers (3 drinks/day), and heavy drinkers (≥ 4 drinks/day) [15]. Active and passive smoking were assessed using pathologically measured cotinine and hydroxycotinine levels, respectively. Sexual behaviors included sexual orientation and the number of sexual partners. Owing to the inclusion and exclusion criteria of this study, the sexual partners of all included participants were women.

Health status included the general health condition, disease history, and other health problems. General health status was determined using a questionnaire. Participants were asked, “Would you say health in general is …?” The answer included 5 levels, which were divided into 3 groups as follows: “Fair” or/and “Poor” were classified as poor health, “Good” was defined as good health, and “Excellent” or/and “Very good” were classified as excellent health. The diseases included hypertension, diabetes, congestive heart failure, coronary heart disease, myocardial infarction, stroke, thyroid problems, kidney failure, kidney stones, and cancer. Hypertension was defined as systolic blood pressure of ≥ 140 mmHg and/or diastolic blood pressure of ≥ 90 mmHg and/or the self‐reported use of anti‐hypertensive medications. Diabetes was defined as a fasting blood glucose level of ≥ 7.0 mmol/L and/or the self‐reported use of anti‐diabetic medications. Health problems included depression, urinary leakage, whether urinary leakage affected daily activities, and circumcision.

Depression severity scores were calculated using the PHQ‐9 scale. The description of each variable can be found at https://www.cdc.gov/Nchs/Nhanes/continuousnhanes/.

### 2.6 Statistical Analysis

Quantitative variables were expressed as the median (interquartile range). These variables were compared using the Mann–Whitney *U* test, as they conformed to a non‐normal distribution. Categorical variables were expressed as the frequencies and percentages and compared using the chi‐square and Fisher tests.

Variables significantly associated with MBAD were identified using three machine learning algorithms (XGBoost, random forest, and LightGBM). Logistic regression analysis was used to construct five models to analyze the association between CMI and MBAD. The crude model was not adjusted for any covariates. Model 1 was adjusted for demographic characteristics (age, race, marital status, and BMI), model 2 was adjusted for lifestyle behaviors (cotinine levels, hydroxycotinine levels, drinking status, and trouble sleeping), model 3 was adjusted for health status (general health condition, hypertension, diabetes, congestive heart failure, coronary heart disease, thyroid problems, myocardial infarction, and kidney stones), and model 4 was adjusted for all of the above mentioned variables. The relationship between CMI and MBAD was further assessed using a generalized additive model (GAM) and restricted cubic spline (RCS) analysis. RCS analysis was also used to examine the relationship between CMI and MBAD in age and BMI subgroups. Age subgroups were classified as < 65 years and ≥ 65 years. Owing to fewer underweight participants in this study, BMI subgroups were classified as < 25 and ≥ 25 kg/m^2^ according to the BMI grouping guidelines established by the World Health Organization (WHO). To validate the association between CMI and MBAD, we converted CMI (a continuous variable) into a categorical variable using quartiles and performed trend regression analysis.

Sensitivity analysis was used to assess the stability of the relationship between CMI and MBAD. Considering the effects of missing data on this relationship, we performed multiple interpolations on missing data using the random forest and *K*‐nearest neighbors (KNN) algorithms and subsequently verified the relationship between CMI and MBAD.

The missForest package in *R* was used to implement random forest, which essentially constitutes a multiple interpolation scheme by averaging over many unpruned classification or regression trees [16]. This approach is more resilient, less affected by outliers, and accommodating to categorical variables. The DMwR package in *R* was used to implement KNN, which interpolates missing data by finding the *K* neighbors closest to the data and using the non‐missing values of the neighbors [17]. KNN‐based interpolation produces data with randomness and uncertainty, yielding good classification accuracy and reliable outcomes. Interaction effect analysis was conducted to assess whether CMI was associated with other variables. All data were analyzed using the SPSS and *R* software. Statistical significance was defined as a *p* value of < 0.05.

## 3 Results

### 3.1 Baseline Characteristics of Study Participants

This study included a total of 1229 participants; of which, 209 participants had MBAD. A total of 29 variables related to demographic characteristics, lifestyle behaviors, and health status were analyzed; among which, 17 variables showed differences between the MBAD and non‐MBAD groups. Compared with the non‐MBAD group, the MBAD group had higher age, BMI, and CMI (median age: 46.00 vs. 56.00; median BMI: 26.70 vs. 30.40; median CMI: 0.47 vs. 0.90) (*p* < 0.001 for all). Table 1 presents the baseline characteristics of participants in the non‐MBAD and MBAD groups.

**TABLE 1:** The basic information of participants in non‐MBAD and MBAD groups.

Variables	Total (*n* = 1229)	Non‐MBAD (*n* = 1020)	MBAD (*n* = 209)	*p*
Age, years	47.00 (32.00, 63.00)	46.00 (31.00, 61.00)	56.00 (41.00, 69.00)	< 0.001
BMI	27.40 (24.00, 30.70)	26.70 (23.70, 30.00)	30.40 (27.10, 36.40)	< 0.001
Sleep duration, hours	7.00 (6.00, 8.00)	7.00 (6.00, 8.00)	7.00 (6.00, 8.00)	0.233
Poverty‐income ratio	2.18 (1.08, 4.25)	2.18 (1.06, 4.28)	2.27 (1.09, 4.10)	0.711
Number of female sexual partners	7.00 (3.00, 16.00)	7.00 (3.00, 16.00)	5.00 (2.00, 20.00)	0.535
Depression score	1.00 (0.00, 3.00)	1.00 (0.00, 3.00)	1.00 (0.00, 4.00)	0.708
CMI	0.51 (0.30, 1.00)	0.47 (0.28, 0.85)	0.90 (0.49, 1.59)	< 0.001
Cotinine, ng/mL	0.06 (0.01, 85.90)	0.06 (0.010, 99.20)	0.03 (0.01, 2.90)	0.002
Hydroxycotinine, ng/mL	0.02 (0.01, 22.70)	0.02 (0.010, 26.50)	0.01 (0.01, 1.85)	0.017
Race, *n* (%)				
Hispanic	269 (21.89)	234 (22.94)	35 (16.75)	0.048
Non‐Hispanic	960 (78.11)	786 (77.06)	174 (83.25)	
Marital status, *n* (%)				
M1	344 (29.58)	308 (32.18)	36 (17.48)	< 0.001
M2	677 (58.21)	543 (56.74)	134 (65.04)	
M3	142 (12.21)	106 (11.08)	36 (17.48)	
Education level, *n* (%)				
Below high school	263 (22.63)	218 (22.78)	45 (21.95)	0.646
High school	268 (23.07)	225 (23.51)	43 (20.98)	
Above high school	631 (54.30)	514 (53.71)	117 (57.07)	
Drinking status, *n* (%)				
Never	124 (10.74)	104 (10.85)	20 (10.26)	0.035
Former	192 (16.64)	150 (15.64)	42 (21.54)	
Light	445 (38.56)	361 (37.64)	84 (43.08)	
Moderate	140 (12.13)	124 (12.93)	16 (8.20)	
Heavy	253 (21.93)	220 (22.94)	33 (16.92)	
Trouble sleeping, *n* (%)				
No	949 (77.22)	799 (78.33)	150 (71.77)	0.039
Yes	280 (22.78)	221 (21.64)	59 (28.23)	
Sexual orientation, *n* (%)				
Heterosexual	748 (96.77)	649 (96.58)	99 (98.02)	0.739
Homosexual	14 (1.81)	13 (1.93)	1 (0.99)	
Bisexual	11 (1.42)	10 (1.49)	1 (0.99)	
General health condition, *n* (%)				
Excellent	247 (21.18)	194 (20.02)	53 (26.91)	0.003
Good	487 (41.77)	396 (40.87)	91 (46.19)	
Poor	432 (37.05)	379 (39.11)	53 (26.90)	
Cancer, *n* (%)				
No	1054 (90.63)	873 (91.22)	181 (87.86)	0.134
Yes	109 (9.37)	84 (8.78)	25 (12.14)	
Stroke, *n* (%)				
No	1126 (96.82)	928 (96.97)	198 (96.12)	0.527
Yes	37 (3.18)	29 (3.03)	8 (3.88)	
Myocardial infarction, *n* (%)				
No	1108 (95.35)	926 (96.76)	182 (88.78)	< 0.001
Yes	54 (4.65)	31 (3.24)	23 (11.22)	
Failing kidneys, *n* (%)				
No	1180 (96.09)	978 (95.976)	202 (96.65)	0.647
Yes	48 (3.91)	41 (4.024)	7 (3.35)	
Kidney stones, *n* (%)				
No	1050 (90.28)	873 (91.22)	177 (85.92)	0.020
Yes	113 (9.72)	84 (8.78)	29 (14.08)	
Hypertension, *n* (%)				
No	763 (65.21)	664 (68.17)	99 (50.51)	< 0.001
Yes	407 (34.79)	310 (31.83)	97 (49.49)	
Diabetes, *n* (%)				
No	918 (74.70)	787 (77.16)	131 (62.68)	< 0.001
Yes	311 (25.30)	233 (22.84)	78 (37.32)	
Congestive heart failure, *n* (%)				
No	1122 (96.56)	928 (97.07)	194 (94.17)	0.039
Yes	40 (3.44)	28 (2.93)	12 (5.83)	
Coronary heart disease, *n* (%)				
No	1104 (95.09)	922 (96.55)	182 (88.35)	< 0.001
Yes	57 (4.91)	33 (3.45)	24 (11.65)	
Thyroid problem, *n* (%)				
No	1121 (96.55)	929 (97.28)	192 (93.20)	0.004
Yes	40 (3.45)	26 (2.72)	14 (6.80)	
Urinary leakage, *n* (%)				
No	892 (81.54)	740 (82.13)	152 (78.76)	0.273
Yes	202 (18.46)	161 (17.87)	41 (21.24)	
Daily activities affected by urinary leakage, *n* (%)				
No	139 (67.15)	106 (67.09)	33 (67.35)	0.973
Yes	68 (32.85)	52 (32.91)	16 (32.65)	
Circumcision, *n* (%)				
No	221 (30.23)	194 (30.65)	27 (27.55)	0.535
Yes	510 (69.77)	439 (69.35)	71 (72.45)	

*Note:* Marital status: M1 (never married, living with partner and separated), M2 (married), and M3 (widowed and divorced).

Abbreviations: BMI, body mass index; CMI, cardiometabolic index.

### 3.2 Determination of Variables Significantly Related to MBAD

The XGBoost, LightGBM, and random forest models were used to assess the relative importance of 17 variables, namely, age, race, BMI, marital status, CMI, cotinine levels, hydroxycotinine levels, drinking status, general health condition, trouble sleeping, hypertension, diabetes, congestive heart failure, myocardial infarction, kidney stones, thyroid problems, and coronary heart disease. Based on the contribution of each variable in the three models, BMI, CMI, and age were identified as the most crucial variables in the dataset (Table 2). These results preliminarily suggested the importance of CMI in MBAD.

**TABLE 2:** The importance assessment of 17 variables on MBAD using the XGBoost, LightGBM, and random forest model.

Variable	Weight importance		
	XGBoost	LightGBM	Random forest
BMI	390	0.266	0.201
CMI	417	0.236	0.197
Age	302	0.235	0.146
Cotinine	215	0.127	0.093
Hydroxycotinine	122	0.041	0.076
Drinking status	94	0.018	0.05
General health condition	60	0.022	0.043
Race	26	0.014	0.021
Hypertension	45	0.000	0.024
Trouble sleeping	36	0.015	0.022
Diabetes	—	0.008	0.023
Marital status	—	0.007	0.032
Congestive heart failure	—	0.006	0.007
Myocardial infarction	—	0.004	0.019
Kidney stones	—	0.000	0.017
Thyroid problem	—	0.000	0.014
Coronary heart disease	—	0.000	0.015

### 3.3 Relationship Between CMI and MBAD

As shown in Table 3, continuous CMI exhibited a notable association with MBAD (crude model: OR = 1.452; 95% CI, 1.282–1.668; model 1: OR = 1.28; 95% CI, 1.139–1.455; model 2: OR = 1.42; 95% CI, 1.252–1.633; model 3: OR = 1.319; 95% CI, 1.168–1.508; model 4: OR = 1.286; 95% CI, 1.134–1.473) (*p* < 0.001 for all). In the fully adjusted model (model 4), age and BMI remained independent risk factors for MBAD (OR_age_ = 1.029; 95% CI, 1.014–1.044; OR_BMI_ = 1.120; 95% CI, 1.087–1.155) (*p* < 0.001 for all).

**TABLE 3:** The association between CMI and MBAD by setting CMI as a continuous variable.

	OR (95% CI)	*p*
Crude model	1.452 (1.282, 1.668)	< 0.001
Model 1	1.280 (1.139, 1.455)	< 0.001
Model 2	1.420 (1.252, 1.633)	< 0.001
Model 3	1.319 (1.168, 1.508)	< 0.001
Model 4	1.286 (1.134, 1.473)	< 0.001

*Note:* Crude model: no variable was adjusted. Model 1: adjusted for age, BMI, race and marital status. Model 2: adjusted for cotinine, hydroxycotinine, trouble sleeping and drinking status. Model 3: adjusted for general health condition, hypertension, diabetes, congestive heart failure, coronary heart disease, thyroid problem, heart attack myocardial infarction and kidney stones. Model 4: adjusted for age, BMI, race, marital status, cotinine, hydroxycotinine, trouble sleeping, drinking status, general health condition, hypertension, diabetes, congestive heart failure, coronary heart disease, thyroid problem, myocardial infarction and kidney stones.

**FIGURE 2:**
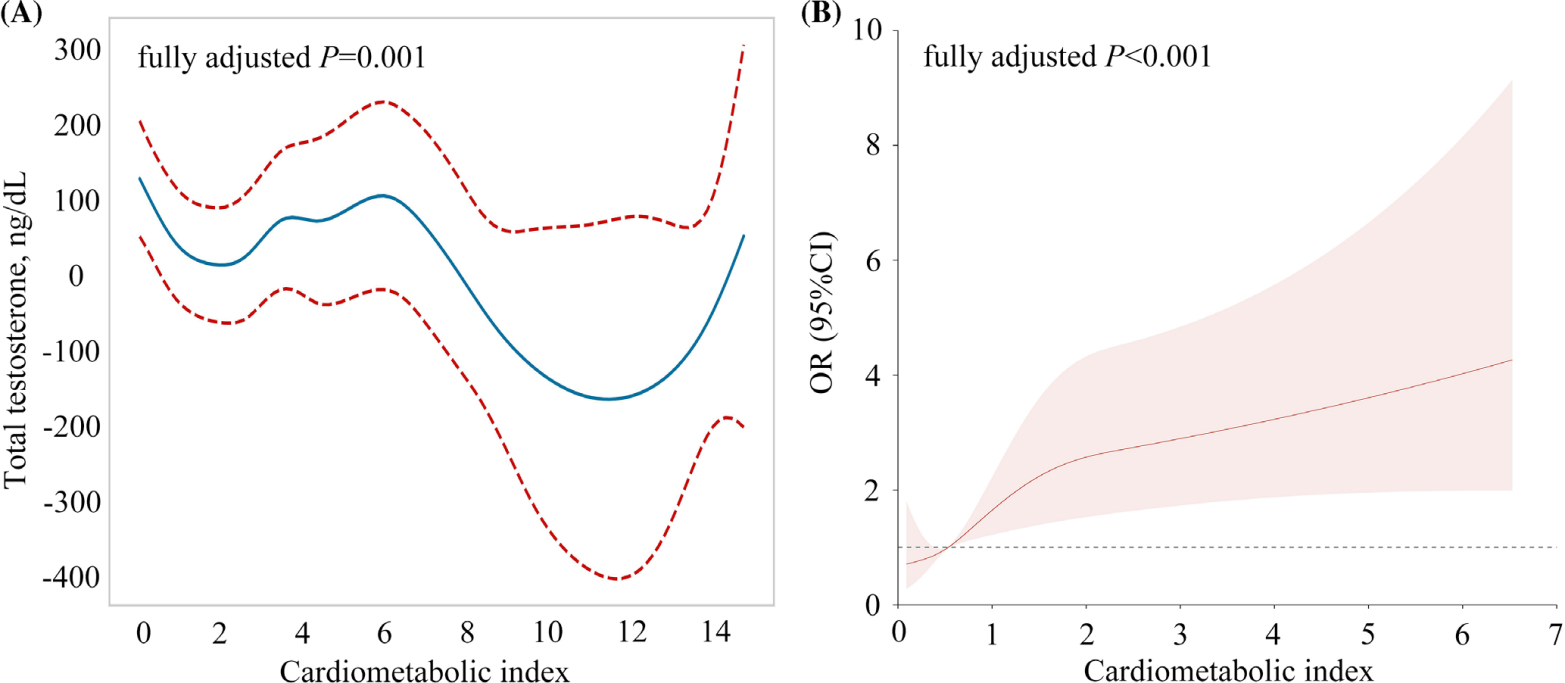
Validation of the relationship between CMI levels and MBAD. (A) Generalized additive model (GAM) analysis. (B) Restricted cubic spline (RCS) analysis. In the GAM and RSC analyses, the age, BMI, race, marital status, cotinine, hydroxycotinine, trouble sleeping, drinking status, general health condition, hypertension, diabetes, congestive heart failure, coronary heart disease, thyroid problem, myocardial infarction and kidney stones were adjusted.

We verified the relationship between CMI and MBAD using a GAM (dependent variable: TT level) and RCS analysis (dependent variable: MBAD) (Figure 2A,B). After adjustments were made for all covariates, both GAM and RCS analysis showed a significant relationship between CMI and MBAD. Specifically, CMI was negatively related to TT levels and positively related to the risk of MBAD (*p*_GAM_ = 0.001; *p*_RCS_ < 0.001). In addition, the OR of MBAD changed at a CMI value close to 0.5378 (from OR < 1 to OR > 1, RCS), indicating the appearance of MBAD risk at the CMI value of > 0.5378.

**FIGURE 3:**
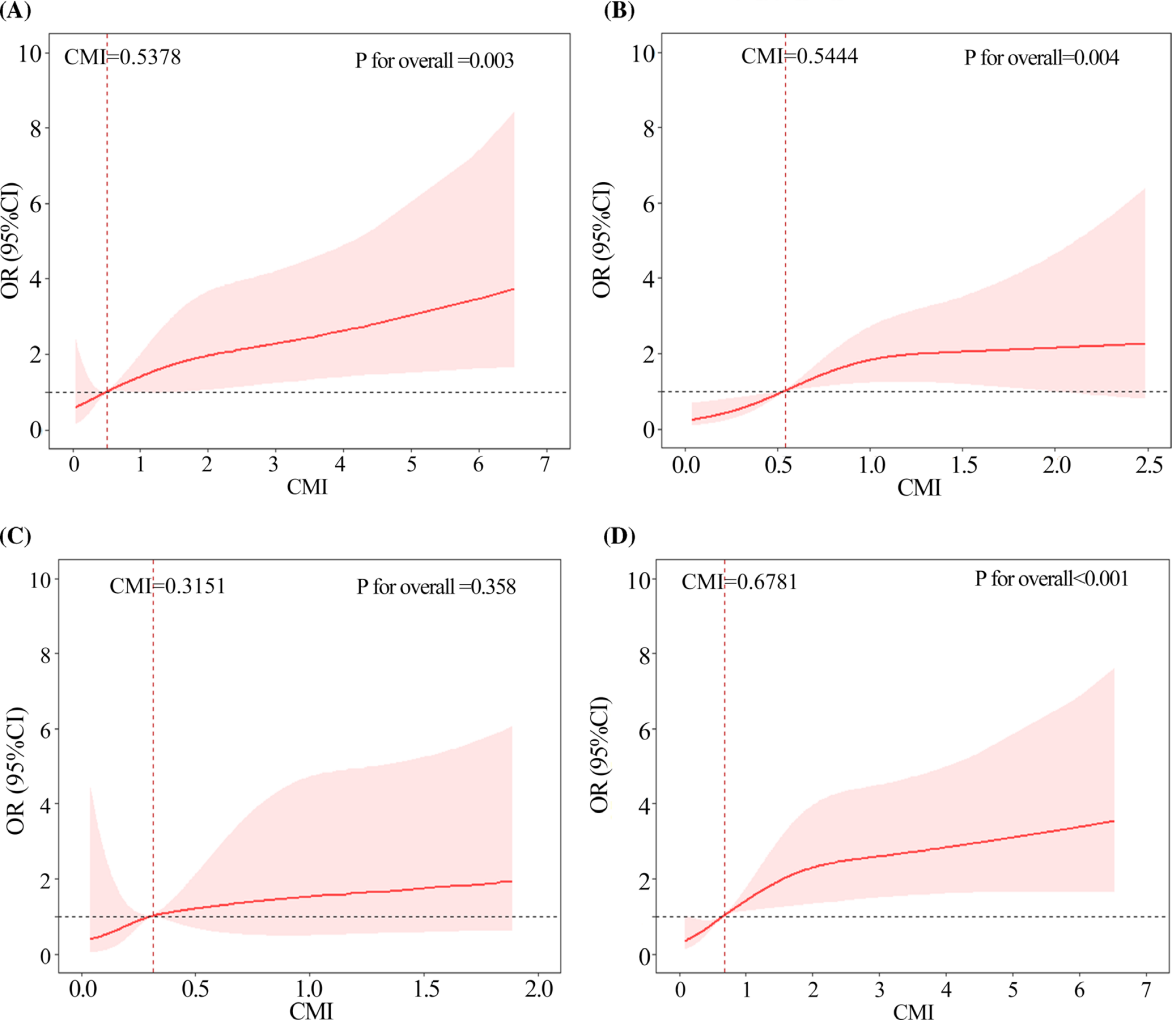
The association between CMI and MBAD in age and BMI subgroup. (A) In age < 65 subgroup. (B) In age ≥ 65 subgroup. (C) In BMI < 25 kg/m^2^ subgroup. (D) In BMI ≥ 25 kg/m^2^ subgroup.

The fully adjusted model (model 4) demonstrated that in addition to CMI, age and BMI were independent factors influencing the risk of MBAD. Furthermore, the relationship between CMI and MBAD was examined in subgroups stratified based on age and BMI. The results indicated that CMI was associated with the risk of MBAD in the age < 65 years and ≥ 65 years subgroups, with changes in the OR of MBAD being observed at CMI values of 0.5378 and 0.5444, respectively (*p* < 0.05 for all) (Figure 3A,B). In the BMI ≥ 25 kg/m^2^ subgroup, participants with a CMI value of ≥ 0.6781 showed a higher probability of developing MBAD (*p* < 0.001). However, the relationship between CMI and MBAD was not statistically significant in the BMI < 25 kg/m^2^ subgroup (Figure 3C,D). It followed that the turning point of CMI relating to MBAD risk appearance was similar between the whole and age‐related subgroup populations but showed slight differences between the whole and BMI‐related subgroup populations.

Subsequently, we conducted trend regression analysis to examine the relationship between changes in CMI and the risk of MBAD (Table 4). The results showed that the OR of MBAD linearly increased with increasing CMI values (crude model: *p* for trend < 0.001) (ORs in the Q1, Q2, Q3, and Q4 groups were 1, 2.271. 3.382, and 6.568, respectively). The positive relationship between MBAD and CMI held in the fully adjusted model (*p* for trend < 0.001), and the OR (95% CI) of MBAD was 2.862 (1.554, 5.270) at the highest CMI (Q4 group) (*p* < 0.001). In the fully adjusted model, the *p* value of OR in the Q3 group (CMI [0.5131, 1.002]) was approximately 0.07, which was close to the threshold of 0.05. These results suggested that participants with a CMI value of > 0.5 had a risk of developing MBAD. Furthermore, we found that the *p* value of the OR of populations with a CMI of > 0.7 was < 0.05, which was statistically significant (data not shown). These findings were similar to those of RCS analysis, suggesting the CMI value at which the risk of MBAD appears.

**TABLE 4:** The influence of CMI changes on the MBAD before and after multiple interpolation.

	Crude model		Fully adjusted model					
			Before multiple interpolation		After multiple interpolation			
					Random forest		KNN	
	OR (95% CI)	*p*	OR (95% CI)	*p*	OR (95% CI)	*p*	OR (95% CI)	*p*
CMI as continuous variable	1.452 (1.282, 1.668)	< 0.001	1.286 (1.134, 1.473)	< 0.001	1.285 (1.142, 1.462)	< 0.001	1.285 (1.143, 1.463)	< 0.001
CMI as categorical variable								
Q1 [0.0409, 0.2967]	1.000 (Reference)		1.000 (Reference)		1.000 (Reference)		1.000 (Reference)	
Q2 [0.2970, 0.5115]	2.271 (1.283, 4.019)	0.005	1.241 (0.651, 2.364)	0.512	1.576 (0.864, 2.872)	0.138	1.563 (0.857, 2.851)	0.145
Q3 [0.5131, 1.0029]	3.382 (1.957, 5.845)	< 0.001	1.755 (0.947, 3.254)	0.074	1.732 (0.953, 3.147)	0.071	1.729 (0.951, 3.142)	0.072
Q4 [1.0031, 14.7057]	6.658 (3.944, 11.241)	< 0.001	2.862 (1.554, 5.270)	0.001	3.082 (1.718, 5.527)	< 0.001	3.087 (1.721, 5.537)	< 0.001
*p* for trend	< 0.001		< 0.001		< 0.001		< 0.001	

*Note:* Crude model: no variable was adjusted. Fully adjusted model: age, BMI, race, marital status, cotinine, hydroxycotinine, trouble sleeping, drinking status, general health condition, hypertension, diabetes, congestive heart failure, coronary heart disease, thyroid problem, myocardial infarction and kidney stones were adjust.

### 3.4 Sensitivity Analysis

To control for the effects of missing values, we used multiple interpolations to fill the missing data. The consistency of the relationship between CMI and MBAD was evaluated before and after multiple interpolations (Table 4). The relationship remained consistent irrespective of the use of the random forest or KNN algorithm. In the fully adjusted model with CMI as the quantitative variable, the ORs (95% CIs) were similar before and after multiple interpolation: pre‐interpolation (OR = 1.286; 95% CI, 1.134–1.473), random forest interpolation (OR = 1.285; 95% CI, 1.142–1.462), and KNN interpolation (OR = 1.285; 95% CI, 1.143–1.463) (*p* < 0.001 for all). In addition, trend regression analysis of CMI as a categorical variable before and after multiple interpolations showed that the OR of MBAD was higher in participants with a CMI value of ≥ 1.0031 (all *p* for trend < 0.001).

On examining the interaction of age and BMI with CMI (Table 5), we found no significant effects of either factor on the relationship between CMI and MBAD (*p* for interaction effect = 0.082 for CMI × age and 0.387 for BMI × CMI).

**TABLE 5:** OR estimates for the association between the CMI and MBAD in age and BMI.

Variables			OR (95% CI)	*p*
MBAD	Interaction 1	Age	1.021 (1.009, 1.033)	< 0.001
		CMI	1.041 (0.706, 1.582)	0.844
		CMI × age	1.008 (0.999, 1.017)	0.082
	Interaction 2	BMI	1.135 (1.097, 1.175)	< 0.001
		CMI	1.739 (0.793, 3.672)	0.152
		BMI × CMI	0.990 (0.968, 1.014)	0.387

The results of sensitivity analysis validated the reliability and stability of the relationship between CMI and MBAD.

## 4 Discussion

TD is a complex endocrine syndrome that is increasingly affecting the aging population. MBAD is considered the pre‐TD stage. In this study, 1229 participants were included from NHANES 2013–2014 and 2015–2016. The prevalence of MBAD in the study population was 17.01% (209/1229 participants). Compared with other studies, which have reported a prevalence of 26.02%–36.4% [3, 18, 19, 20], this study showed a lower prevalence of MBAD, which may be attributed to differences in study populations or inclusion and exclusion criteria.

CMI has been associated with various endocrine conditions, such as diabetes mellitus, infertility, and endometriosis [11, 21, 22]. In this study, CMI was found to be associated with the risk of MBAD after adjustments were made for other variables. RCS analysis revealed a positive relationship between CMI and MBAD, which remained consistent in age and BMI subgroups. The cut‐off values of RCS varied between subgroups but were very similar. These values were similar to the cut‐off values observed in the Q3 (CMI: 0.5131, 1.0029) group in trend regression analysis (although the *p* value of the Q3 group was 0.07, it was close to 0.05). Trend regression analysis suggested that participants with a CMI value of ≥ 1.0031 had a higher risk of developing MBAD. This relationship remained consistent after multiple interpolations, indicating the robustness of the findings. These results suggest that CMI is a risk factor for MBAD and that close monitoring of CMI may help prevent the development of MBAD.

Studies have shown a correlation between BMI and serum testosterone levels, with patients with obesity exhibiting lower testosterone levels [23, 24]. More importantly, obesity is considered to be associated with TD [25], with asymptomatic TD being referred to as MBAD. In this study, subgroup analysis showed that CMI was associated with MBAD in overweight or obese populations and in the entire adult male population. However, interaction analysis showed that neither BMI nor age had significant effects on the relationship between CMI and MBAD, suggesting the high stability of the relationship.

In this study, a high CMI (CMI ≥ 1.0031) was identified as a risk factor for MBAD, suggesting that CMI might influence the occurrence of MBAD. As CMI serves as a novel and comprehensive measure of obesity and lipid metabolism, we propose the following hypothesis to explain the relationship between CMI and MBAD. Adipose tissue releases cytokines such as IL‐1β, TNF‐α, and IL‐6, which suppress the hypothalamic–pituitary–testicular (HPT) axis, resulting in reduced production of testosterone [26]. Dyslipidemia is characterized by increased levels of TG‐rich lipoproteins and decreased levels of HDL‐C [27]. High levels of TGs, LDL‐C, and TC lead to oxidative stress, which can negatively affect male reproductive function and may cause MBAD by directly or indirectly affecting the HPT axis and decreasing testosterone levels [28, 29]. Aromatase (CYP19A1) in adipose tissue converts testosterone to estradiol (E2), decreasing beneficial testosterone levels and promoting fat accumulation. The increased estrogen exerts negative feedback on the hypothalamus, inhibiting the secretion of gonadotropin‐releasing hormone (GnRH) [30]. Excess estrogen leads to underproduction of luteinizing hormone (LH) and follicle‐stimulating hormone (FSH) for steroidogenesis and induces systemic inflammation, negatively affecting the production of testicular steroids [31]. Obesity can increase leptin secretion from adipose tissue [32]. High leptin levels cause central leptin resistance in the hypothalamic–pituitary gland, inhibiting the secretion of GnRH and LH and reducing the sensitivity of Leydig cells to LH [33]. The relationship between CMI and MBAD appears to be influenced by multiple pathways; however, the precise underlying mechanism warrants further investigation.

Both CMI and TD are related to many diseases. This study demonstrated a positive relationship between CMI and the risk of MBAD, the pre‐TD stage. Future studies should focus on investigating the mediatory role of CMI in the relationship between MBAD and other diseases or the mediatory role of MBAD in the relationship between CMI and other diseases.

Despite valuable findings, this study has some limitations that should be acknowledged. First, serum TT levels were measured only once in NHANES, whereas the relevant guidelines recommend that the levels should be measured twice. Second, the relationship between CMI and MBAD was analyzed via retrospective analysis, and the causal relationship between the two factors remains unknown. Third and last, CMI data were available for only 21.18% of adult male participants included in this study (NHANES 2013–2016). Therefore, studies with large sample sizes are required to validate the relationship between CMI and MBAD.

## 5 Conclusion

This study revealed a positive relationship between CMI and the risk of MBAD through various statistical methods. Additionally, sensitivity analysis validated the stability of this relationship. In conclusion, the findings of this study suggest that CMI is a risk factor for MBAD and that close monitoring of CMI may help prevent the development of MBAD. Future studies should focus on validating the causal relationship between CMI and the risk of MBAD.

## Conflicts of Interest

The authors declare no conflicts of interest.

## Data Availability

The datasets generated during and/or analyzed during the current study are available from the corresponding author upon reasonable request.
